# Internet Search Patterns of Human Immunodeficiency Virus and the Digital Divide in the Russian Federation: Infoveillance Study

**DOI:** 10.2196/jmir.2936

**Published:** 2013-11-12

**Authors:** Andrey Zheluk, Casey Quinn, Daniel Hercz, James A Gillespie

**Affiliations:** ^1^Menzies Centre for Health PolicyThe University of SydneyUniversity of Sydney NSWAustralia; ^2^PRMA Consulting LtdNew York, NYUnited States; ^3^University of SydneySydneyAustralia

**Keywords:** Russia, search engine, human immunodeficiency virus, surveillance

## Abstract

**Background:**

Human immunodeficiency virus (HIV) is a serious health problem in the Russian Federation. However, the true scale of HIV in Russia has long been the subject of considerable debate. Using digital surveillance to monitor diseases has become increasingly popular in high income countries. But Internet users may not be representative of overall populations, and the characteristics of the Internet-using population cannot be directly ascertained from search pattern data. This exploratory infoveillance study examined if Internet search patterns can be used for disease surveillance in a large middle-income country with a dispersed population.

**Objective:**

This study had two main objectives: (1) to validate Internet search patterns against national HIV prevalence data, and (2) to investigate the relationship between search patterns and the determinants of Internet access.

**Methods:**

We first assessed whether online surveillance is a valid and reliable method for monitoring HIV in the Russian Federation. Yandex and Google both provided tools to study search patterns in the Russian Federation. We evaluated the relationship between both Yandex and Google aggregated search patterns and HIV prevalence in 2011 at national and regional tiers. Second, we analyzed the determinants of Internet access to determine the extent to which they explained regional variations in searches for the Russian terms for “HIV” and “AIDS”. We sought to extend understanding of the characteristics of Internet searching populations by data matching the determinants of Internet access (age, education, income, broadband access price, and urbanization ratios) and searches for the term “HIV” using principal component analysis (PCA).

**Results:**

We found generally strong correlations between HIV prevalence and searches for the terms “HIV” and “AIDS”. National correlations for Yandex searches for “HIV” were very strongly correlated with HIV prevalence (Spearman rank-order coefficient [*rs*]=.881, *P*≤.001) and strongly correlated for “AIDS” (*rs*=.714, *P*≤.001). The strength of correlations varied across Russian regions. National correlations in Google for the term “HIV” (*rs*=.672, *P*=.004) and “AIDS” (*rs*=.584, *P*≤.001) were weaker than for Yandex. Second, we examined the relationship between the determinants of Internet access and search patterns for the term “HIV” across Russia using PCA. At the national level, we found Principal Component 1 loadings, including age (-0.56), HIV search (-0.533), and education (-0.479) contributed 32% of the variance. Principal Component 2 contributed 22% of national variance (income, -0.652 and broadband price, -0.460).

**Conclusions:**

This study contributes to the methodological literature on search patterns in public health. Based on our preliminary research, we suggest that PCA may be used to evaluate the relationship between the determinants of Internet access and searches for health problems beyond high-income countries. We believe it is in middle-income countries that search methods can make the greatest contribution to public health.

## Introduction

### Search Patterns in Health

Internet search patterns provide a low-cost, rapidly accessible data source for a range of health problems. Search patterns have been described as behavioral measures of an issue’s importance to individuals [1]. If individual Internet users are concerned or interested in an issue, they are more likely to search for information related to that issue. The relative importance of an issue among populations of Internet users can thus be inferred from the volume of searches for a term or terms representing that issue. Since 2006, researchers have used search patterns to study a wide range of health problems, notably influenza [2-5], as well as undocumented adverse drug interactions [6,7], suicide-related information [8], and HIV (human immunodeficiency virus) [9]. Despite the widespread use of search patterns, researchers commonly suggest Internet users may not be representative of the entire population. Specific concerns include differences in access based on age [10], income and education [11], and gender [12]. This means the social, economic, and demographic status of Internet users may not fully reflect those of the population as a whole.

### Determinants of Internet Access

The propensity to access the Internet varies between socioeconomic and demographic cohorts. The strongest determinants of Internet access are income and education. This finding is consistent in studies from the United States [13] and the European Union [14] and across middle-income countries [15]. Additionally, gender [16], English-language ability [17], broadband access price [18], urban location [19], ethnicity [20], and age [21] have also been reported as determinants of Internet use in both high- and middle-income countries. In summary, Internet users in both high- and middle-income countries are more likely to have higher incomes and higher levels of education.

### The Digital Divide—Access and Use

Access to the Internet is an economic development policy issue. Telecommunications networks, including the Internet, are regarded as a catalyst for economic growth [22]. Since the early 2000s, the term “digital divide” has been widely used to describe differences in Internet access and use across socioeconomic gradients within and between countries [23]. In 2011, Hilbert reviewed international policy responses to the digital divide [24]. In his review, Hilbert proposes four classes of variables with which to analyze the digital divide. These classes are the unit of analysis (eg, individual, country), determinants of access (eg, income, education), the kind of technology (eg, cell phones, fixed broadband), and how individuals connect (ie, access vs effective use). Others have similarly argued that access to infrastructure inadequately describes the digital divide [25]. Basing their arguments on Roger’s theory of diffusion of innovations, these authors suggest analysis of the digital divide should focus on effective use, incorporating technical competence, and individuals’ adaptation of technology to meet their personal needs rather than access alone.

### Use of the Internet for Health Information Seeking Online

The Internet is widely used for health information seeking in high-income countries. A 2013 study found 59% of all US adults searched for health information online, with 77% of these starting at search engines such as Google [26]. Equally, there is a general scholarly consensus that a digital divide applies to online health seeking behavior [27]. In 2006, Rice described the limited research into health-related Internet use across economic and demographic gradients in the United States [28]. More recent studies European [29] and US [30] studies suggest that income and education are the most important determinants of seeking health information online.

### Search Patterns and Effective Use

Although a digital divide may exist, determining the sociodemographic profile of Internet users from search results is not straightforward. Aggregated Google search queries are the most commonly used data source for search studies but carry no demographic or economic information. In the case of disease surveillance, this means that groups with a significant disease burden, such as older or economically disadvantaged people without Internet access may be excluded from search results [31]. By contrast, health information seeking research is generally based on qualitative research and statistical surveys. This research generally includes demographic characteristics and covers issues such as health literacy [32] and behaviors following access to health information [33]. In summary, researchers have widely investigated the effective use of online health information in high-income countries. It is this research that provides the empirical foundation for a rich analysis of the relationship between health information seeking across economic and demographic gradients and patterns of online search.

### Chronic Illness and Internet Use

Individuals with chronic health problems and disabilities are more likely to search for health information online. Online information seeking among people with chronic and terminal diseases has been widely researched [34,35]. Cancer information seeking in particular has attracted considerable research interest due to its diversity, duration, and treatment complexity [36]. The management of HIV as a chronic illness has similarly attracted scholarly interest. Studies suggest PLHIV (people living with HIV) use the Internet extensively for health information. A 2006 US study found that 66% of PLHIV participants searched for health information at least half the time they were online [37]. Furthermore, PLHIV Internet users were more likely to be better educated, have higher incomes, exhibit greater knowledge of HIV disease processes, and adhere to medication [38,39]. In summary, while income and education are the most important determinants of health-related Internet use, individuals with chronic diseases may have a stronger incentive to use the Internet effectively.

### Online Health Information Seeking in Middle-Income Countries

While research is limited, online health information seeking also appears to be important in middle-income countries. In 2011, the international health insurer Bupa surveyed online health information seeking among Internet users in 12 high- and middle-income countries [40]. The researchers found higher rates of health information seeking in middle income countries (China 94%, Thailand 93%, and Saudi Arabia 91%) than in high-income countries (Australia 77%, United Kingdom 70%, and Spain 71%). Similarly, a 2010 Bupa study found 95% of Russian Internet users sought advice on health, medicines, or medical conditions online [41]. Bupa researchers attributed the high rates of online health information seeking in middle-income countries to the high cost of medical consultations and concerns over service quality. While not peer reviewed, these Bupa surveys point to a particularly important role for health-related searches outside of high-income countries. Conversely, these studies investigated only the propensity to access health information among Internet users, leaving aside international comparisons of how effectively online health information is used across social and economic gradients. The relationship between the need for health information and access to the Internet was not investigated.

### Search Studies in Middle-Income Countries

As recently as 2009, researchers suggested that Google Trends was unsuitable for disease surveillance outside of developed countries due to insufficient Internet access [42]. However, the rapid increase of Internet use in middle-income countries suggests otherwise. Internet use is forecast to grow considerably more quickly by 2015 in middle-income than high-income countries (see Table 1; [43]). Since 2009, studies from Southeast Asia [44], Latin America [45], Russia [46], and China [47] suggest that search pattern studies are increasingly regarded as valid and reliable methods of disease surveillance in middle-income countries.

The potential of search patterns to improve public health surveillance in middle-income countries is well documented. First, online surveillance offers immediate insights into the present status of disease. That is, online surveillance may “predict the present” [48] without the reporting lags associated with complicated reporting procedures in public health bureaucracies [44]. Second, online surveillance may overcome the weaknesses of traditional surveillance systems, such as poor sensitivity to new diseases [49] and the lack of skills and equipment required for early disease detection [50]. Third, searches may overcome underreporting gaps from the private sector and from individuals who do not seek formal medical care [51]. Fourth, online surveillance may improve transparency. Central or regional governments may wish to minimize reports of disease outbreaks that could affect tourism or political reputation [52] or what sensitive issues surveys may not reveal [53,54]. In summary, online surveillance has the potential to improve disease surveillance in populations bearing the greatest burden of disease.

Consistent with the aims of infodemiology, our exploratory study examined “the science of distribution and determinants of information...(on) the Internet, or in a population, with the ultimate aim to inform public health and public policy” [55]. We examined the relationship between Internet search patterns, disease prevalence, and the determinants of Internet access using the case of HIV in a middle-income country. Through investigating these relationships, we aimed to develop methods to complement traditional HIV surveillance in Russia and contribute to the science of health-related searches.

**Table 1:** Changes in Internet use in selected middle- and high-income countries (values indicate penetration in %, ie, number of users divided by population).

Country	2009 actual Internet use	2015 predicted Internet use
China	28	47
India	7	19
Brazil	33	74
Russia	31	55
Indonesia	12	37
United States	70	73
Japan	74	81

## Methods

### Summary

This exploratory study sought to determine if search methods can be used for disease surveillance in a large middle-income country with a dispersed population. We first assessed whether online surveillance is a valid and reliable method for monitoring HIV in the Russian Federation. Second, we analyzed the determinants of Internet access to determine the extent that they explain regional variations in searches for the Russian terms for “HIV” and “AIDS”.

### Google and Yandex Searches in Russia

Most search pattern studies have used Google Trends (or the defunct Google Insights for Search) as the data source. Google Trends has been deployed in studies of influenza [3], dengue [11], and HIV [9]. However, the structure of the Russian-language Internet market is unique. Whereas Google provided 84% of global Internet search queries in May 2011 [56], Google’s market share in Russia was only 25% in 2010/2011 [57]. The largest search provider in Russia in 2011 was Yandex, with 60% market share. In 2011, Russia overtook Germany as the European country with the highest number of unique visitors online [58]. Russian Internet users grew from 43% of the population in 2010 to 55% in 2012 [59]. In Russia, Yandex is a strong commercial competitor of Google.

The publicly available Google Trends data for Russia has several limitations. First, Google does not provide complete results, returning only subregions with the highest search volume. Google data were available for only 16 of Russia’s 89 subregions for the term “HIV” and 29 for the term “AIDS” during 2011. Second, Google does not provide raw search data. This makes direct comparisons between subregions and matching with variables representing Internet access determinants complex. We used WordStat as the primary data source, as Yandex made publicly available a complete raw search dataset for all Russian regions and subregions for the full 12 months of 2011. We used Google Trends as a secondary source of aggregated search results for validation purposes.

### Case Study: Why is Search-Based HIV Surveillance Important in Russia?

HIV is a serious health problem in the Russian Federation. Russia has the highest cumulative number of PLHIV of any European country, largely concentrated among people who inject drugs (PWID). On December 31, 2011, there were 650,100 PLHIV registered in Russia [60]. However, the true scale of HIV in Russia has long been the subject of considerable debate [61,62]. Feshbach and colleagues’ 2005 study compiled data from official and unofficial Russian sources, as well as international agencies, to assess the quality of Russian HIV statistics [63]. The authors suggested that official Russian HIV data are frequently inconsistent, diverge markedly from alternative sources such as UNAIDS (the Joint United Nations Programme on HIV/AIDS), and present major methodological obstacles. The authors concluded that official Russian estimates of HIV prevalence were understated by a multiple of three to five times. Similar findings emerged from a 2007 UNODC (United Nations Office on Drugs and Crime) report that evaluated national data collection mechanisms related to HIV among PWIDs in nine lower income countries including Russia [64].

### HIV Surveillance and Hidden Populations

HIV surveillance is further complicated by Russian drug laws, police, medical, and public attitudes. Most international observers regard Russian drug laws as punitive, unsupported by scientific evidence, and ineffective [65]. A 2010 study into police behavior found widespread reports of extrajudicial policing practices, including extortion, torture, and rape of PWIDs [66]. Attitudes among medical staff too are generally negative towards PLHIV [67,68]. Public opinion is also generally negative towards individuals acquiring HIV sexually or through drug use [69]. As a consequence of professional and social attitudes, many PLHIV avoid contact with medical organizations and avoid testing for HIV. Literature suggests there are disincentives for Russian PLHIV accessing health information directly from health professionals.

International researchers generally regard Russian HIV-positive PWIDs as a population hidden from public health surveillance. Since the early 2000s, researchers have sought to improve population estimates and document the conditions experienced by Russian PWIDs living with HIV [70,71]. Traditional surveys and sampling methods among PWIDs are unreliable, as individuals may not report accurately on stigmatized and illegal behaviors. A 2011 study in Russia found that, among 193 HIV-positive participants, only 36% were aware of their HIV status [72]. Another study of HIV-positive Russian PWIDs found persistent high-risk behaviors associated with HIV transmission [73]. Among study participants, 25% had been refused access to medical care, 18% were refused employment or fired, and 6% were forced from family homes. Researchers found 39% of participants had probable clinical depression, and 37% had anxiety levels comparable to psychiatric inpatients. In summary, there is considerable evidence that Russia has large numbers of PLHIV, many of whom are likely to be alienated from the formal health system and be absent from official statistics. The high rates of Internet searches for health information, combined with stigmatization of HIV, suggest that the Internet may be an important resource for PLHIV in Russia.

Russian injecting drug users have generally avoided contact with the formal health system. Between 2004 and 2011, much of the contact with injecting drug users and other groups at high risk of HIV was conducted by donor-funded Russian non-governmental organizations (NGOs). The behavioral surveillance data collected by these NGOs also contributed to Russian national HIV reporting to UNAIDS. However, as the result of government pressures, the number of donor-funded harm reduction NGO projects in Russia decreased from 70 in 2007 to 20 in 2011 [74]. The decrease in NGOs may also have eroded the capacity for data collection from populations at risk of HIV. In 2012, Russia did not report any HIV behavioral surveillance data associated with injecting drug use and sex work [75]. In summary, the progressive dismantling of harm reduction projects in Russia means only surveillance data from individuals formally diagnosed with HIV in government clinics are available. Injecting drug workers, sex workers, and others at risk of HIV have disappeared from Russian government reporting.

### RQ1 Method: Is Search Surveillance a Valid Method for Monitoring HIV in Russia?

To answer this research question, we examined the relationship between HIV prevalence across the Russian Federation and Internet searches for the terms “HIV” and “AIDS”. First, we obtained HIV prevalence data for each region and subregion from the Russian Federal AIDS Centre [60]. We chose 2011 data as this was latest complete dataset available. The Russian Federal AIDS Centre publishes the most timely and comprehensive HIV dataset available. However, these data are limited to formally diagnosed PLHIV and likely exclude many individuals at risk of HIV, or of uncertain serostatus, who deliberately avoid contact with government health services.

Second, we selected two terms to represent HIV searches. These two search terms were “HIV” (VICh in Russian) and “AIDS” (SPID). We referred to the Google Trends related-terms feature [76] to ensure each term referred to the subject of this study. In the case of “HIV”, all terms were related to HIV, whereas the term “AIDS” revealed several unrelated terms (Table 2). For example, the second most popular term associated with “AIDS” referred to the computer game “need for speed”. Based on these results, we anticipated that the search term “HIV” would have a stronger positive correlation with HIV prevalence than the term “AIDS”.

Third, we aggregated Yandex searches for each month of 2011 to produce a single annual search figure for the terms “HIV” and “AIDS” for each of Russia’s 89 subregions (see the map in Multimedia Appendix 1; [77]) covering the data range January 1 to December 31, 2011. In Russian federal statistical compilations, several smaller subregions are routinely aggregated, producing 83 rather than 89 statistical subregions. In our calculations, we used population prevalence of HIV and searches rather than raw figures. This allowed comparison across regions and subregions. We used this single 2011 annual HIV search in our further calculations.

Fourth, we conducted Spearman correlations of per-capita Yandex monthly searches for the terms “HIV” and “AIDS” against HIV prevalence data for all Russian subregions in 2011. We repeated this process with each of eight Russian regions. This provided us with national and regional correlations between search and prevalence data for 2011.

Fifth, we obtained all available Google data for the terms “HIV” and “AIDS” for 2011 and repeated this analysis for validation purposes. Google search data for the term “HIV” were available for 16 regions and for “AIDS” for 29 regions. We then conducted Spearman correlations between Google and Yandex data for validation purposes.

**Table 2:** Google Trends—Related terms for HIV and AIDS in the Russian Federation in 2011.

Search related terms		Russian	Value
**“HIV”**			
	symptoms HIV	симптомы вич	100
	symptoms	симптомы	100
	AIDS	спид	65
	AIDS HIV	спид вич	65
	HIV infection	вич инфекции	35
	HIV signs	вич признаки	35
	analysis for HIV	анализ на вич	35
	HIV infection	вич инфекция	30
	HIV dating	вич знакомства	25
	HIV photo	вич фото	20
**“AIDS”**			
	test AIDS	тест спид	100
	need for speed	нид фор спид	75
	AIDS HIV	спид вич	55
	HIV	вич	55
	AIDS info	спид инфо	50
	AIDS centre	спид центр	45
	AIDS symptoms	спид симптомы	25
	AIDS photo	спид фото	25
	AIDS test	спидтест	25
	speed hack	спид хак	20

### RQ2: What is the Relationship Between the Determinants of Internet Access and Searches for the Term “HIV” Across Russia?

The relationship between Internet search patterns for specific health problems and the prevalence of these problems in populations is now well established. However, Internet users may not be representative of overall populations. Further, the characteristics of the Internet using population cannot be directly ascertained from search pattern data. We sought to extend understanding of the characteristics of Internet searching populations through data matching the determinants of Internet access (ie, age, income, broadband access price, and urban to rural ratios) with search patterns through multivariate analysis.

Several studies have examined the socioeconomic factors associated with HIV prevalence and injecting drug use in Russia. Moran et al investigated the relative importance of several variables in influencing HIV prevalence in a cross-sectional study based solely on Russian federal government statistics [78]. The authors found urbanization, mobility, crime, and income growth associated with HIV prevalence. In 2011, researchers surveyed 711 PWIDs in two large provincial cities [79]. The researchers concluded PWIDs were typical Russians when compared with a random population. However, investigators drew their random sample from 2004 household survey data. While Russian per capita income grew from US $9800 in 2004 to US $17,000 in 2011 [80], the authors did not comment on this potentially important confounder. These two studies illustrate the logistic difficulties of obtaining timely, valid, and independent data in Russia.

### Our Methodology

We examined the relationship between spatial patterns of online searches for the term “HIV” and the determinants of Internet access. We used data from RQ1 in our analysis. In RQ1, we demonstrated the relationship between HIV prevalence and searches for “HIV”. While this relationship was generally strong, differences in search patterns across regions may reflect differences in the determinants of Internet access as well as differences in HIV rates.

We selected principal component analysis (PCA) to explore the relationship between the determinants of Internet access and searches for the term “HIV”. PCA is a method of multivariate analysis for finding patterns in data rather than hypothesis testing. PCA aids in the interpretation of relationships in the original data by transforming the original variables into a new set of variables, the principal components [81,82]. PCA has been widely used in public health to study relationships of health problems to socioeconomic variables. For example, PCA has been used to investigate European tumor prevalence [83], nutritional epidemiology in Greece [84], and epidemiological analysis in low- and middle-income countries [85]. As a consequence, we considered PCA an appropriate method for this exploratory study.

First, we collated the data sources. We obtained search pattern data for the term “HIV” through PCA. We obtained Russian-language data for five determinants of Internet access (see Table 3) from the Russian federal statistics agency for 83 Russian statistical regions [86]. The determinants of Internet access comprised each a single figure for each subregion for 2011. In compiling our data, we sought to most closely align search pattern, HIV prevalence, and determinants of Internet access data.

Second, we conducted PCA on all Russian subregions to produce a national level analysis. We included the determinants of Internet access and per capita search for “HIV” for all subregions. We used a correlation matrix approach to standardize the variables, as we used different units with differing variances. Based on our review of Internet determinants, we anticipated that the variables we chose to analyze would correlate.

Third, we conducted separate PCAs to examine the relationship between HIV search patterns and the determinants of Internet access separately on each of the eight Russian regions. Previous research suggests that, while there is no minimum of variables and cases in PCA, a larger number is preferable [87]. In designing this study, we purposely selected a smaller number of variables. We did this to permit analysis of both national data, as well as of regions with smaller number of subregions. Through analyzing both national and regional PCA separately, we anticipated we would identify additional spatial relationships not obvious at the national level.

**Table 3:** Determinants of Internet access—List of variables in PCA.

Variable	Determinant of Internet access (abbreviation)
Variable 1	Higher education students per 100,000 population (age)
Variable 2	Percentage aged 25-64 with higher education (education)
Variable 3	Gross regional product per capita (income)
Variable 4	Broadband price per month (Bband price)
Variable 5	Urban / rural population (urbanization)
Variable 6	Searches for HIV per 100,000 population during 2011 (search)

## Results

### Correlations

We first investigated search surveillance as a valid method for monitoring HIV in Russia. We found generally strong correlations between HIV prevalence and searches for the terms “HIV” and “AIDS”. Yandex searches for “HIV” were very strongly correlated with HIV prevalence (Spearman rank-order coefficient [*rs*]=.881, **P*≤.*001), whereas “AIDS” was strongly correlated nationally (*rs*=.714, *P≤*.001) (see Table 4). The strength of correlations varied across Russian regions. Several regions were less strongly correlated in Yandex. For example, HIV prevalence and searches in the central and northwestern regions were moderately correlated as a result of outlier data points. Further, Google national searches for the term “HIV” were moderately correlated (*rs*=.672, *P*=.004) with HIV prevalence and weakly correlated with Yandex searches for “HIV” (*rs*=.584, *P≤*.001) (see Table 5).

Second, we examined the relationship between the determinants of Internet access and search patterns for the term “HIV” across Russia. We found considerable variation in the relationship between these determinants and search patterns. We first analyzed national PCA results (Table 6). We determined the number of components to analyze using the Kaiser, Scree, and cumulative variance methods [82]. Kaiser and scree tests suggested three principal components (PCs), and the cumulative variance method suggested four PCs should be analyzed. In PC1 (the first and most important component), HIV search, age, and educational variables were moderately correlated. In PC2, per capita income was most important. This factor was weakly correlated with searches for HIV and explained 23% of the variance. The subsequent two components, which explain less variance, are more difficult to interpret.

**Table 4:** HIV and AIDS correlations from Yandex—National and all federal regions of Russian Federation.

Region	HIV prevalence per 100,000 pop’n	Searches for “HIV” per 1000 pop’n	Spearman correlation for HIV prevalence & “HIV” (2-tailed *P* value)	Searches for “AIDS” per 1000 pop’n	Spearman correlation for HIV prevalence & term “AIDS” (2-tailed *P* value)
National	446.513	16.995	.881 (≤.001)	19.312	.714 (≤.001)
Central	279.2	21.832	.377 (.006)^a^	21.215	-.123 (.386)
Northwestern	586.6	23.619	.482 (≤.001)^a^	26.383	.209 (.137)
Southern	144.3	8.397	.486 (≤.001)	12.665	.486 (≤.001)
North Caucuses	58.8	2.758	-.179 (.206)	6.666	-.286 (.040)
Volga	437.8	17.322	.793 (≤.001)	20.366	.380 (.005)
Urals	805	24.037	.657 (≤.001)	19.379	.429 (≤.001)
Siberian	528.1	13.962	.804 (≤.001)	15.561	.503 (≤.001)
Far east	166.4	7.473	.017 (.907)	11.197	.083 (.557)

^a^Outliers.

**Table 5:** Google Trends search results.

	AIDS searches	HIV searches
Number of regions	29	16
Spearman correlation, HIV prevalence (2-tailed *P* value)	.584 (*P≤*.001)	.672 (*P*=.004)
Spearman correlation, Google with Yandex (2-tailed *P* value)	-.289 (*P*=.129)	.223 (*P*=.406)

**Table 6:** National PCA results.

Importance of components		PC1	PC2	PC3	PC4	PC5	PC6
Standard deviation		1.386	1.172	0.989	0.854	0.737	0.674
Proportion of variance		0.320	0.229	0.163	0.121	0.091	0.076
Cumulative proportion		0.320	0.549	0.712	0.834	0.924	1.000
**Loadings**							
	Age	-0.560	–	0.105	0.269	0.762	0.121
	Education	-0.479	-0.376	0.313	0.247	-0.345	-0.593
	Income	0.215	-0.652	–	-0.491	0.460	-0.268
	Bband price	0.329	-0.460	0.523	0.422	–	0.478
	Urbanization	0.131	-0.417	-0.777	0.396	-0.110	0.190
	Search	-0.533	-0.200	–	-0.539	-0.278	0.546

### Biplots and Spatial Relationships

**Figure 1:**
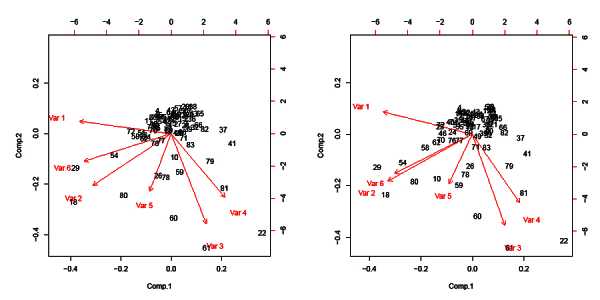
National HIV search and HIV prevalence biplots.

We used biplots to explain spatial relationships in our PCA results. Biplots provide a visual representation of PCA data from the first two PCs [88]. Biplots allow identification of clusters of subregions with similar characteristics. Further, the clustering of subregions along vector lines serves to highlight subregions more strongly associated with specific variables. Importantly, the clustering of subregions is subjective and requires additional analysis. On the national HIV search biplot (see Figure 1), PC1 was associated with Vector 3 (income), Vector 4 (broadband price), and Vector 5 (urbanization). PC2 was associated with Vector 1 (age), Vector 2 (education), and Vector 6 (HIV search). We obtained PCA results and biplots for all eight Russian regions. See Multimedia Appendix 2 for a list of subregions referenced in the national PCA. See Multimedia Appendices 3-5 for biplot results for each Russian federal region. Finally, we conducted a separate PCA for HIV prevalence data. We substituted the variable HIV search with HIV prevalence. The results of a PCA incorporating the variable HIV prevalence produced results with a similar form to those incorporating HIV searches at both the national and regional levels.

**Table 7:** Summary of national biplots.

Region	Relationship	Geographic clusters	Outliers
National	PC1: V3, V4, V5 Income, broadband fees, urbanization	Cluster 1 37. Ingushetia 41. Chechnya 79. Amursk 81. Sakhalin 83. Chukhotka	18. Moscow City 22. Nenets Autonomous Region 29. St Petersburg 61. Yamalo-Nenets
		Cluster 2 10. Moscow region 71. Kemerovo 26. Murmansk 78. Khabarovsk 59. Tyumen	
	PC2: V1, V2, V6 Age, education, and HIV prevalence/ search	Cluster 3 18. Moscow city 24. Kaliningrad 29. St Petersburg City 46. Tatarstan 54. Samara 58. Sverdlovsk 62. Chelyabinsk 70. Irkutsk 76. Kamchatka 77. Primorsk 80. Magadan	

## Discussion

### Principal Findings

Overall, we found search patterns were a valid method of HIV surveillance in the Russian Federation. Furthermore, our research suggests that search patterns for HIV are generally not related to income or broadband price. However across Russian regions, we found considerable variation in the strength of correlations between search and disease prevalence, and the determinants of Internet access. Finally, our analysis suggested that the strong correlations between search and disease prevalence may indicate effective use of the Internet by individuals at risk of HIV and PLHIV.

### RQ1: Is Search Surveillance a Valid Method for Monitoring HIV in Russia?

We found online search patterns for HIV were correlated with HIV prevalence in both Google and Yandex at the national level. It is noteworthy that the latest official Russian HIV data available at the time of writing in mid-2013 were for the year 2011. By contrast, Yandex search data were available with a delay of 4 weeks and Google data with a 48-hour delay. This timely availability illustrates the potential contribution of search pattern data to disease surveillance.

Second, we found considerable variation in the strength of correlations among regions in Yandex data. Overall, we found Yandex searches for the term “HIV” and HIV prevalence were most strongly correlated. This suggests PLHIV are more likely to search for “HIV” than “AIDS”. In the North Caucuses and far eastern regions, HIV prevalence was not positively correlated with search. We attribute this to the low HIV prevalence and low search volumes in these regions. By contrast, in the central and northwestern regions, search volumes and HIV prevalence were high, but correlations were moderate. We attributed the weaker correlations to outliers in Yandex data. Removing the central (Moscow subregion) and northwestern region (Leningrad subregion), outliers strengthened correlations from 0.377 to 0.551 and 0.482 to 0.939 respectively. This suggests correlation analysis should routinely account for outlying subregions.

Third, we found Google data are not adequate for subnational HIV surveillance in Russia. We attribute the low correlations to the multiple zero values present in our Google dataset. Of the 15 regions for which Google data were available, many months recorded a zero search value for the term “HIV”. These zero values were consistent with an earlier study [46] of the use of Google search for health policy analysis in Russia that found Google Trends requires an unknown threshold before results are displayed. While national level Google data were correlated with HIV prevalence, our analysis suggests it should not be used for regional analysis.

Finally, our results contribute to understanding of hidden populations of PLHIV in Russia. There is a general consensus that Russian HIV rates are underreported. Previous studies have reported considerable at-risk populations unaware of their HIV status in subregions with high HIV prevalence (eg, [74]). However, we found strong spatial correlations between official HIV rates and searches for HIV. This finding has several interpretations. First, the spatial variation in search results also appears in traditional surveillance. Researchers have found high populations of unknown HIV serostatus in subregions of high HIV prevalence. That is, additional searches for HIV related information by populations at risk of HIV and unknown serostatus may inflate already high search volumes in those subregions with high HIV prevalence. However, these additional searches would not change the overall spatial distribution of search patterns. A second interpretation relates to the search data used. Our analysis relied on annually aggregated search results. Our results are thus a static view of HIV prevalence over a 12-month period. This static view does not capture longitudinal anomalies in search patterns. While this 12-month snapshot was appropriate for the purposes of this study, monitoring of weekly and monthly search patterns may produce different results and reveal spatial variations in searches.

### RQ2: What is the Relationship Between the Determinants of Internet Access and Search Patterns for the Term “HIV” Across Russia?

We analyzed national and regional PCA results separately. First, we examined the two national level biplots. One biplot incorporated HIV prevalence as a variable and the other searches for “HIV”. All other variables remained consistent (see Figure 1). We found these two biplots to be near isomorphic.

The separate national biplots containing both HIV prevalence and HIV search produced three logically coherent geographical clusters. National cluster 1 was characterized by low-income, non-ethnic Russian subregions with low HIV prevalence. The exception to this is the Sakhalin subregion, with high per capita income. This clustering occurred along the broadband vector (V4), suggesting high broadband prices and limited access. National cluster 2 was associated with the urbanization vector (V5). It includes urbanized non-metropolitan areas. National cluster 3 included the Russian cities with the highest prevalence of HIV, along the HIV search/prevalence (V6) and education vectors (V2). In addition, it included the Magadan subregion. The Magadan subregion is highly urbanized but has a low HIV prevalence and low population. We attribute the inclusion of Magadan in this cluster as an indicator of potential HIV risk. Conversely, the isolation of Magadan, in northeastern Russia, away from borders and drug routes, suggests a lower risk of HIV transmission through injecting drug use.

Both national PCA biplots featured several outlier subregions (see Figure 1). For example, the Yamalo Nenets subregion (61) was an outlier. This is an oil-producing subregion, with very high per capita incomes and below average HIV rates. It was strongly associated with the income vector (V3). Second, Moscow and St Petersburg were outliers. We attribute these cities’ outlier positions to a statistical anomaly. Each city had a rural to urban ratio of zero and very high Internet access rates and incomes. In summary, national level PCA analysis of both the HIV prevalence and HIV search biplots suggested a stronger relationship between broadband access prices in several subregions. See Multimedia Appendix 4 for a discussion of PCA in Russian regions.

In summary, PCA is not a technique that establishes causal relationships. However, based on our preliminary analysis, we suggest that income and broadband prices do not generally appear to be associated with HIV searches, either positively or negatively, in the subregions of highest HIV prevalence. Further research, in the form of confirmatory factor analysis and regression analysis is needed to establish this relationship statistically. Contingent upon the results of this additional analysis, HIV search pattern data may be incorporated into HIV modeling.

Our findings extend beyond an examination of the digital divide in Russia as defined by access to the Internet. There is also a behavioral dimension implicit in our two research questions. Search patterns measure aggregate behavior at the population level, with important issues more frequently searched. Searches for the term “HIV” measure the importance of this disease in a population. Consequently, the generally strong correlations between search patterns and disease prevalence lead us to infer that the Internet is being used effectively by PLHIV. That is, searchers for “HIV” demonstrate the technical competence to search for health information they consider important. However, this is a cautious conclusion, and one that merits further research.

### Further Research and Limitations

Our research suggests that further exploratory analysis applying search pattern methods to HIV surveillance in Russia is warranted. First, PWID and sex worker populations may be at increased risk of HIV as the result of the Russian government’s censorship of prevention, treatment, and care information [89,90] and decreased behavioral monitoring capacity among internationally funded NGOs. Further, in 2013, concerns emerged about the capacity of independent Russian social research organizations to continue unencumbered data collection [91]. Search methods may present a partial solution to these emerging information constraints. Internet search patterns provide a valid near real time measure of health behaviors in the field at population level.

Second, additional research is required to establish how effectively Russians use the Internet for HIV and health information. Qualitative and survey research among populations at risk of HIV and PLHIV will assist the further development of search surveillance methods and the planning of online interventions. Research in Russia should also examine the quality of health information available to PLHIV, both through domestic and international Russian language websites.

Third, organizations working with at-risk populations and PLHIV may consider initiating studies that establish baseline measures of search patterns for HIV and related diseases. From these baselines, longitudinal studies will be able to rapidly identify unanticipated shifts in spatial and temporal patterns of HIV-related searches and HIV prevalence, well in advance of official incidence and prevalence data.

Fourth, the method described in this paper can be extended to other communicable and non-communicable diseases in Russian-speaking countries. Broader application of this method may require initial disease-by-disease and country-by-country validation. However, even without validation, this method provides a low-cost, rapid, timely initial assessment with which to shape further planning, analysis, and decision making.

Finally, our research had several limitations. First, we were constrained by the absence of time series data. To conduct data matching for the PCA, we used a single aggregate figure to represent total searches nationally and within each subregion. We believe this analysis would be strengthened by a month-to month comparison of HIV prevalence data in each Russian region. Such data are not publicly available. Second, Google is the only data source in most middle income countries. This limits the application of this method. An important exception is China, where Baidu is increasingly being used alongside Google for disease surveillance.

### Conclusions

The use of data for disease surveillance has been widely promoted in popular literature. Under the rubric of “big data”, journalists have popularized the novel application of Internet search patterns in medicine [92]. Scholars too, have speculated that data availability will lead to the evolution of new models of disease surveillance [93-95]. While the potential application of large scale data analysis in health care has generated considerable popular and scholarly interest, most research has focused on high-income countries with well-functioning public health information systems. We believe it is in middle-income countries that search methods can make the greatest contribution to public health. It is in these countries that traditional surveillance systems are least developed and health data least available.

Clearly, a digital divide between rich and poor countries persists. However, Internet access in middle-income countries is growing rapidly, and online health information is in demonstrably high demand. Based on our preliminary research, we are cautiously optimistic in suggesting that access to the Internet should therefore not be considered a constraint to conducting search studies beyond high-income countries. It is in lower income countries that search pattern surveillance may move beyond a statistical novelty and be incorporated into local health data collection and decision making.

## Multimedia Appendix 1

Map of Russian Federation.

## Multimedia Appendix 2

Reference list of Russian regions and subregions for PCA biplots.

## Multimedia Appendix 3

Narrative analysis of PCA in Russian regions.

## Multimedia Appendix 4

PCA biplots - Russian regional HIV search and HIV prevalence.

## Multimedia Appendix 5

Table summarizing PCA biplot results in Russian regions.

## Abbreviations

AIDS: Acquired Immune Deficiency Syndrome
HIV: Human Immunodeficiency Virus
NGO: non-governmental organization
PC: principal component
PCA: principal components analysis
PLHIV: people living with HIV
PWID: people who inject drugs
UNAIDS: Joint United Nations Programme on HIV/AIDS
UNODC: United Nations Office on Drugs and Crime
