# Correction: Internet Search Patterns of Human Immunodeficiency Virus and the Digital Divide in the Russian Federation: Infoveillance Study

**DOI:** 10.2196/jmir.3112

**Published:** 2013-11-26

**Authors:** Andrey Zheluk, Casey Quinn, Daniel Hercz, James A Gillespie

**Affiliations:** ^1^ Menzies Centre for Health Policy The University of Sydney University of Sydney NSW Australia; ^2^ PRMA Consulting Ltd New York, NY United States; ^3^ University of Sydney University of Sydney NSW Australia

The order of references in “Internet Search Patterns of Human Immunodeficiency Virus and the Digital Divide in the Russian Federation: Infoveillance Study“ (J Med Internet Res 2013 (Nov 12); 15(11):e256) was incorrect. References 60-95 were out of order, while references 1-59 were correct and remain unchanged. Specifically, reference 60 should have been deleted, and references 61-76 shifted up one to become references 60-75. Reference 78 is now 76, while reference 77 remains unchanged. References 79-87 are now shifted up one to become references 78-86, while references 88-95 remain unchanged. Reference 87 is replaced with a new reference (Jolliffe).

This error has been corrected in the online version of the paper on the JMIR website on November 26, 2013, together with publishing this correction notice. A correction notice has been sent to PubMed. This was done before submission to Pubmed Central and other full-text repositories.

