# Supplementary figures and images for: Internet Search Patterns of Human Immunodeficiency Virus and the Digital Divide in the Russian Federation: Infoveillance Study
Source: J Med Internet Res. 2013 Nov 12;15(11):e256. doi: 10.2196/jmir.2936 (PMC3841350; doi:10.2196/jmir.2936)

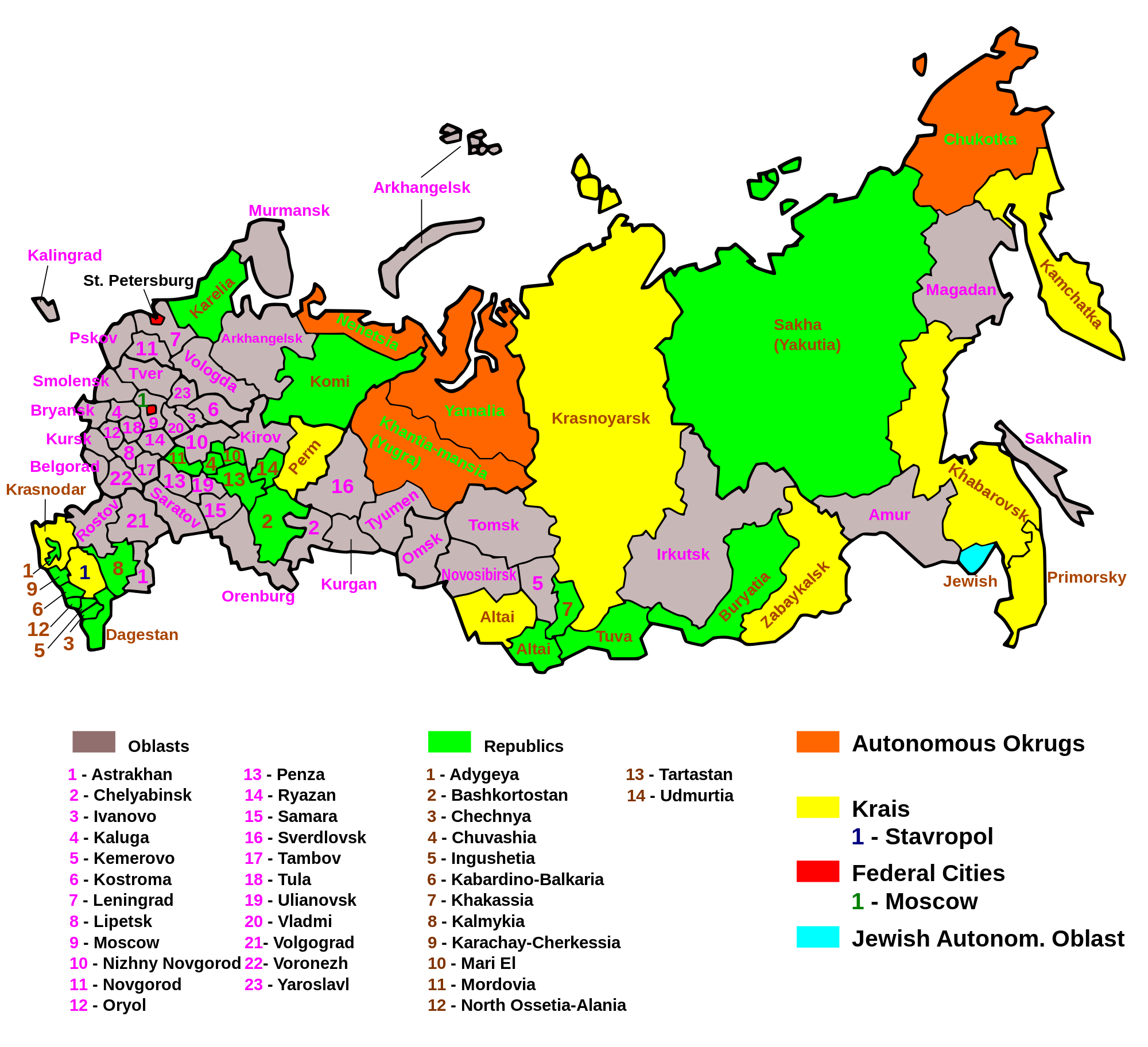

Supplement: Supplementary file 1 [file jmir_v15i11e256_app1.png]
