# Supplementary material for: Internet Search Patterns of Human Immunodeficiency Virus and the Digital Divide in the Russian Federation: Infoveillance Study
Source: J Med Internet Res. 2013 Nov 12;15(11):e256. doi: 10.2196/jmir.2936 (PMC3841350; doi:10.2196/jmir.2936)
Supplement: Supplementary file 2 [file jmir_v15i11e256_app2.pdf]

List of regions and subregions reference for PCA biplots

Name	National PCA	Regional PCA
**Central**		**A**
Belgorod	1	A1
Bryansk	2	A2
Vladimir	3	A3
Voronezh	4	A4
Ivanovo	5	A5
Kaluga	6	A6
Kostroma	7	A7
Kursk	8	A8
Lipetsk	9	A9
MoscowReg	10	A10
Orel	11	A11
Ryazan	12	A12
Smolensk	13	A13
Tambov	14	A14
Tver	15	A15
Tula	16	A16
Yaroslavl	17	A17
MoscowCity	18	A18
**North Western Federal Region**		**B**
Karelia	19	B1
Komi	20	B2
Archangelsk	21	B3
Nenets Autonomous Region	22	B4
Vologda	23	B5
Kaliningrad	24	B6
Leningrad	25	B7
Murmansk	26	B8
Novgorod	27	B9
Pskov	28	B10
St. Petersburg City	29	B11
**Southern Federal Region**		**C**
Adygia	30	C1
Kalmykia	31	C2
Krasnodar	32	C3
Astrakhan	33	C4
Volgograd	34	C5
Rostov	35	C6
**North Caucuses Federal Region**		**D**
Dagestan	36	D1
Ingushetia	37	D2
KabardinoBalkaria	38	D3
KarachayevoCherkessiya	39	D4
NorthernOssetiya	40	D5
Chechnya	41	D6
Stavropol	42	D7

**Volga Federal Region**		**E**
Bashkortostan	43	E1
MariyEl	44	E2
Mordovia	45	E3
Tatarstan	46	E4
Udmutriya	47	E5
Chuvashiya	48	E6
Perm	49	E7
Kirov	50	E8
Nizhegorod	51	E9
Orenburg	52	E10
Penza	53	E11
Samara	54	E12
Saratov	55	E13
Ulyanovsk	56	E14
**Urals Federal Region**		**F**
Kurgan	57	F1
Sverdlovsk	58	F2
Tyumen	59	F3
Khanty-Mansiysk	60	F4
Yamalo-Nenetsk	61	F5
Chelyabinsk	62	F6
**Siberian Federal Region**		**G**
Altay Republic	63	G1
Buryatiya	64	G2
Tyva	65	G3
Khakasiya	66	G4
Altay Region	67	G5
Zabaykal	68	G6
Krasnoyarsk	69	G7
Irkutsk	70	G8
Kemerovo	71	G9
Novosibirsk	72	G10
Omsk	73	G11
Tomsk	74	G12
**Far East Federal Region**		**H**
Sakha (Yakutiya)	75	H1
Kamchatka	76	H2
Primorsk	77	H3
Khabarovsk	78	H4
Amursk	79	H5
Magadan	80	H6
Sakhalin	81	H7
Jewish Autonomous Region	82	H8
Chukhotka	83	H9
