# Supplementary material for: Internet Search Patterns of Human Immunodeficiency Virus and the Digital Divide in the Russian Federation: Infoveillance Study
Source: J Med Internet Res. 2013 Nov 12;15(11):e256. doi: 10.2196/jmir.2936 (PMC3841350; doi:10.2196/jmir.2936)
Supplement: Supplementary file 3 [file jmir_v15i11e256_app3.pdf]

Appendix 4 - Narrative analysis of PCA in Russian regions

This Appendix provides a narrative analysis of the main features of PCA analysis from the Russian regions. We observed considerable variation between Russian regions in the relationships between the determinants of internet access and use (Appendix 4A-H and 5-A-H) In analysing regions, we primarily focused our analysis on the relationships with HIV search/prevalence. As in the case of national PCA, we clustered subregions within each region, and analysed both HIV prevalence and search in two separate biplots. We found logically coherent clusters of subregions within regions, associated with specific variables. For example, in the Urals region (F), the Sverdlovsk subregion was most closely associated with both the HIV search and HIV prevalence vector (V6) In 2011 Sverdlovsk had the second highest HIV prevalence in Russia [53], suggesting our PCA biplots produced results consistent with alternative data sources.

In the Far East (H), we found two subregions clustered around the broadband vector. These two subregions, Amursk and Jewish Autonomous subregions, had high broadband prices(Vector4). Also in the Far East region, we identified a cluster of most two subregions most strongly associated with HIV prevalence/search These were the Khabarovsk and Primorsk subregions. Khabarovsk had almost double the broadband price of Primorsk, a considerably lower HIV prevalence (128.5 vs 348.6) and a similar per capita rate of internet search for HIV. This may indicate undiagnosed PLHIV in Khabarovsk. Conversely, it may indicate exogenous factors such local school projects or news reports stimulating internet search. However the small population, low search volumes and low HIV prevalence in most subregions suggest results from the Far East should be treated with caution.

We found outlier regions required active analysis and incorporating incidence data. For example in the Siberia Region (G), the Kemerevo subregion (subregion 9) was an outlier not associated with any vectors. In 2011, Kemerovo had the highest incidence of HIV of all Russia subregions [53]. This suggests incidence data should also be included in PCA analyses. Similarly, in the Volga region (Table 4A), no subregion was strongly associated with the HIV prevalence/ HIV search vector. However, an outlier, the Samara subregion (12) had the highest regional incidence of HIV, while another outlier, Perm (7) had the second highest. These outliers were not evident in the national level PCA.

Finally, in the North Caucasus region (D), we identified three geographical clusters. For example, Cluster 2, Chechnya and Ingushetia, had the lowest per capita incomes in Russia, and the highest prevalence of HIV in the North Caucasus in 2011 [53]. However, these two subregions also had among the highest broadband price in Russia and recorded low search rates for HIV. These low search rates, combined with high broadband prices, relatively high subregional HIV rates suggest the provision of HIV prevention, treatment and care may be a priority in these parts of the Northern Caucuses.
