# Supplementary material for: Internet Search Patterns of Human Immunodeficiency Virus and the Digital Divide in the Russian Federation: Infoveillance Study
Source: J Med Internet Res. 2013 Nov 12;15(11):e256. doi: 10.2196/jmir.2936 (PMC3841350; doi:10.2196/jmir.2936)
Supplement: Supplementary file 4 [file jmir_v15i11e256_app4.pdf]

National and Regional biplots

List of variables in PCA

Variable1: Higher Education Students per 100000 population {Age)
Variable2: Percentage of aged 25-64 with Higher Education (Education)
Variable3: Gross Regional product per capita (Income)
Variable4: Broadband price per month (Bband price)
Variable5: Urban / rural Population (Urbanisation)
Variable6: Searches for HIV per 100000 population during 2011 (Search)

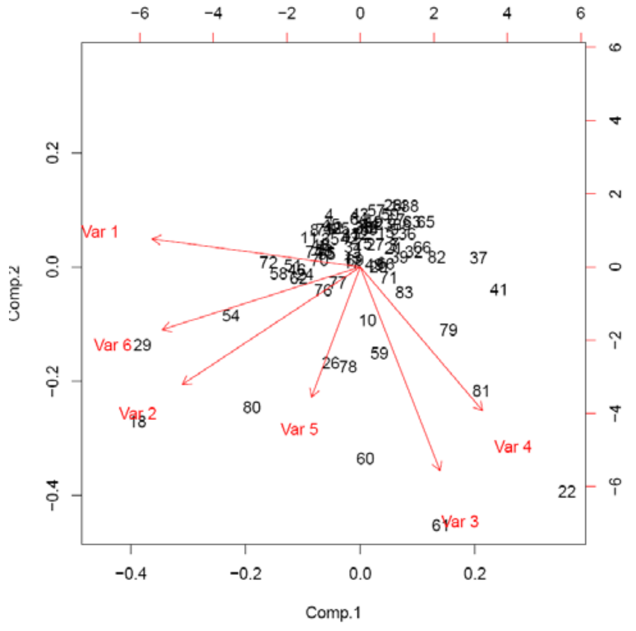

National - HIV Search biplot

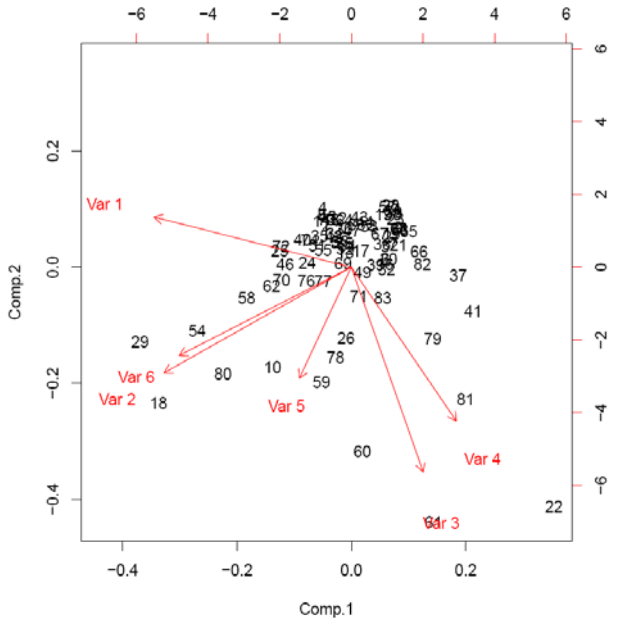

National - HIV Prevalence biplot

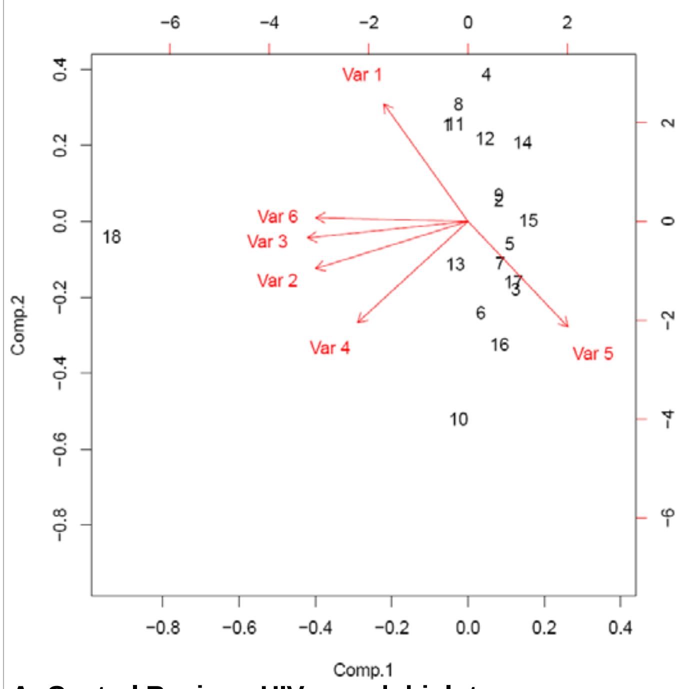

A: Central Region - HIV search biplot

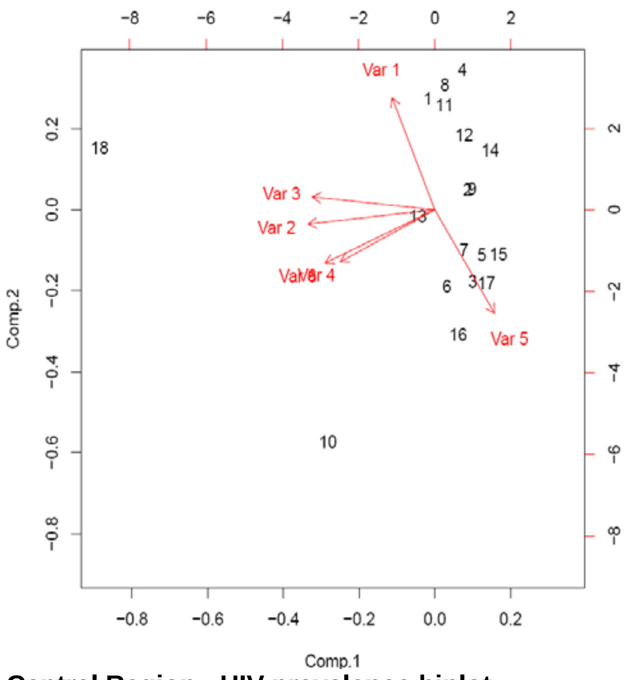

A: Central Region - HIV prevalence biplot

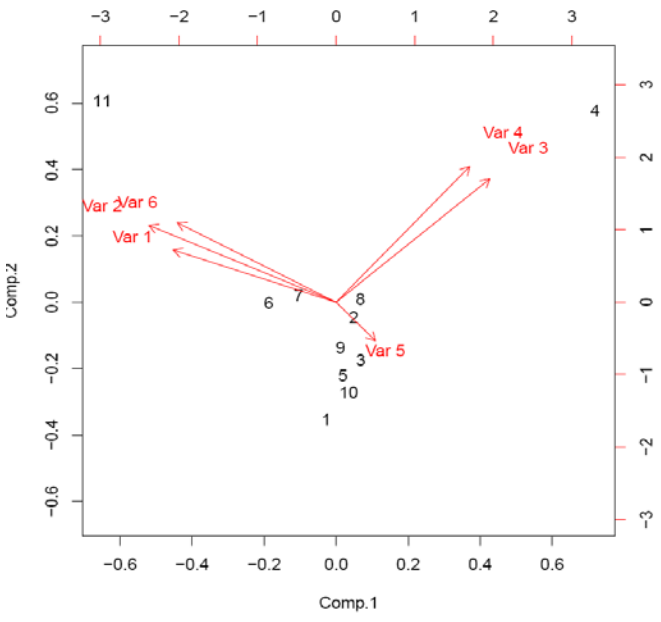

B: North West region- - HIV search biplot

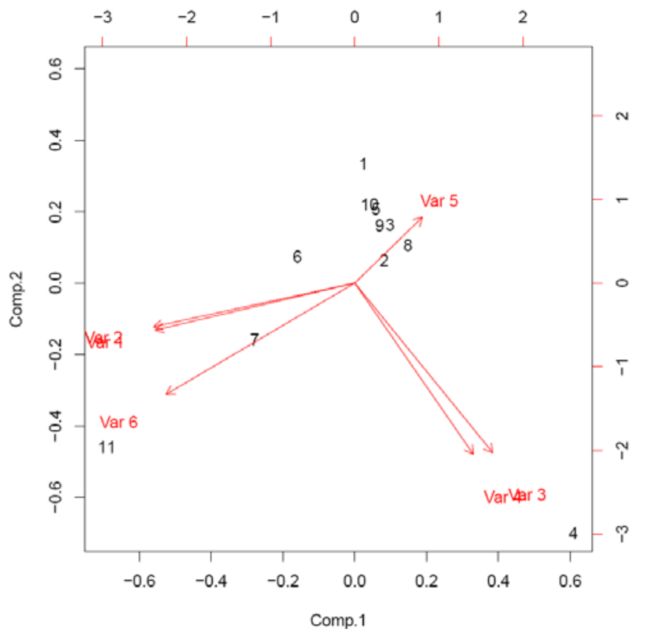

B North West region HIV prevalence biplot

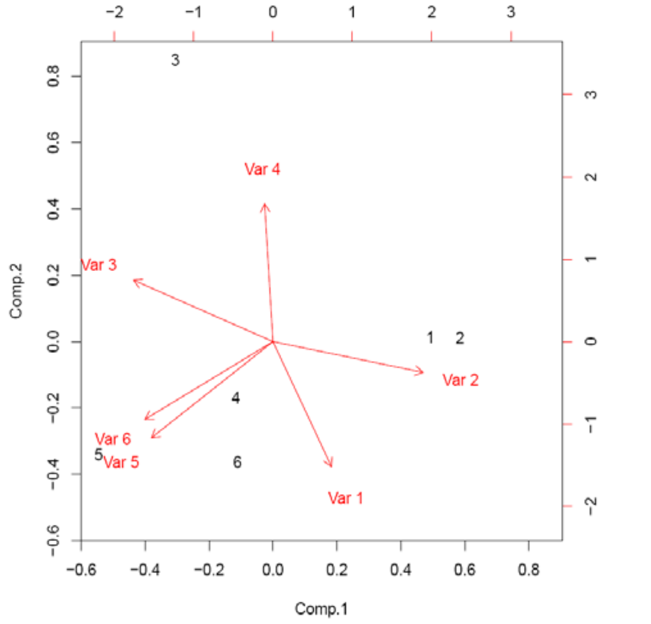

C: Southern Region - HIV Search biplot

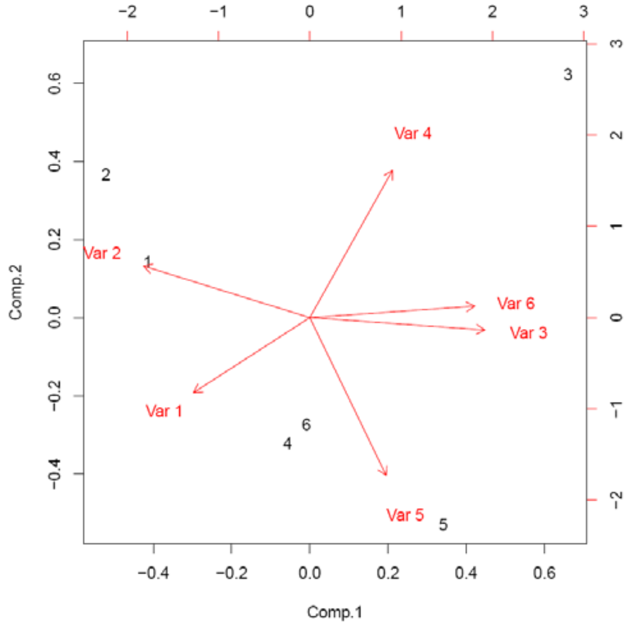

C: Southern Region - HIV prevalence biplot

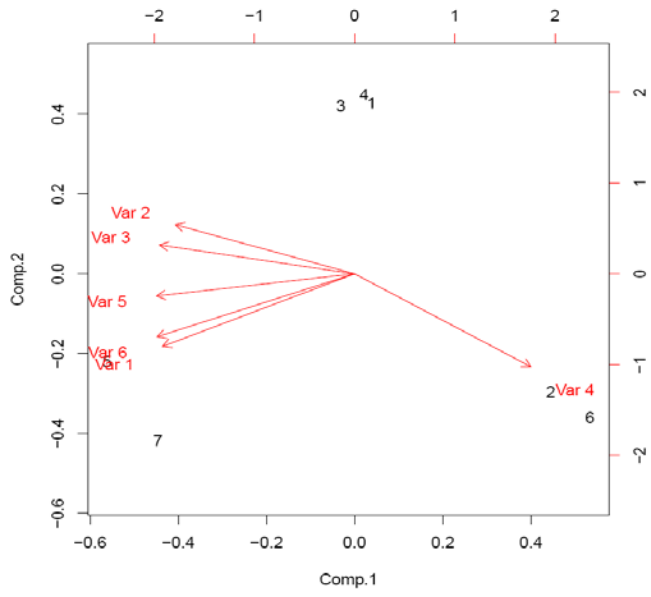

D North Caucuses Region HIV search biplot

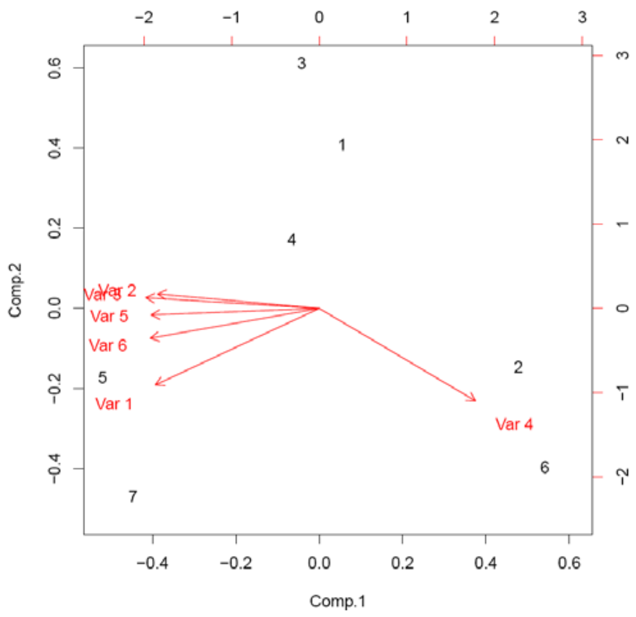

D North Caucuses HIV Prevalence biplot

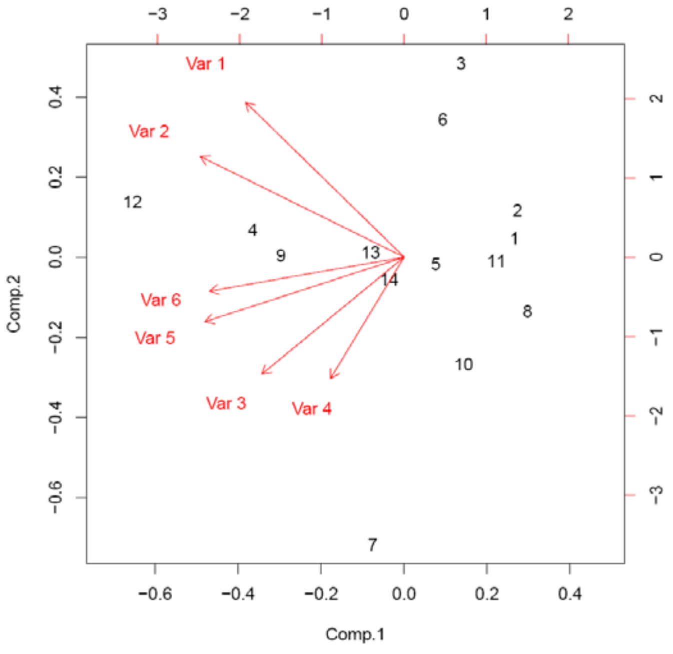

E Volga Region HIV search biplot

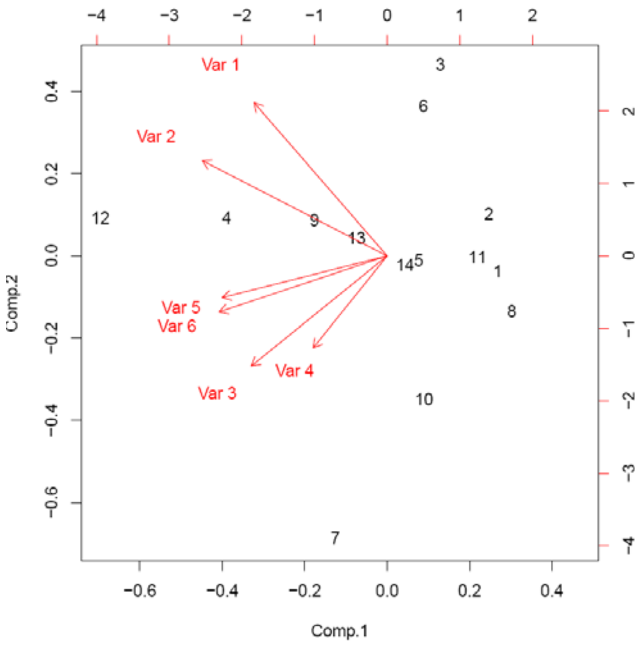

E Volga Region HIV prevalence biplot

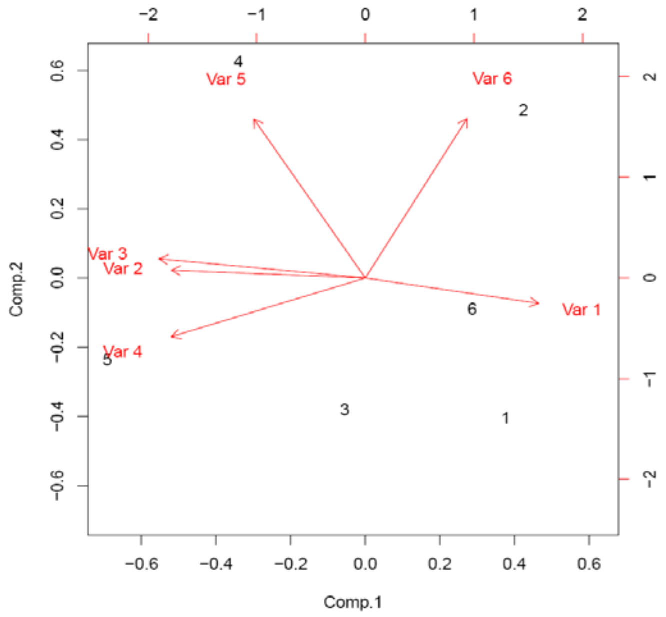

F Urals Region HIV search biplot

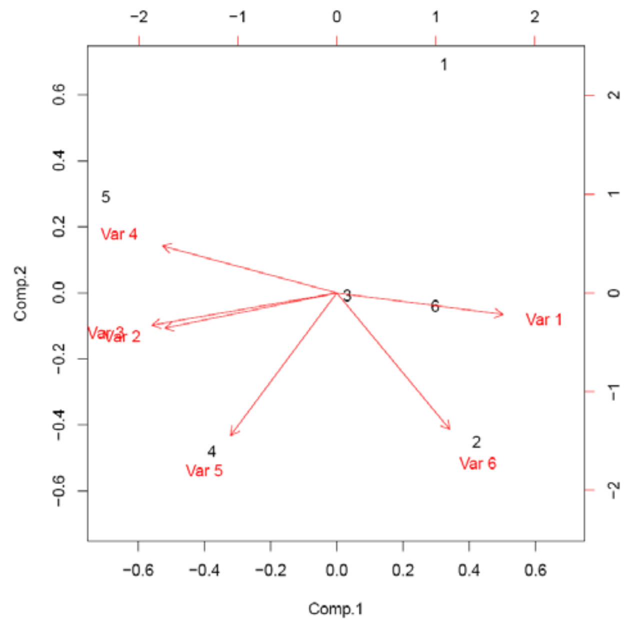

F Urals Region HIV prevalence biplot

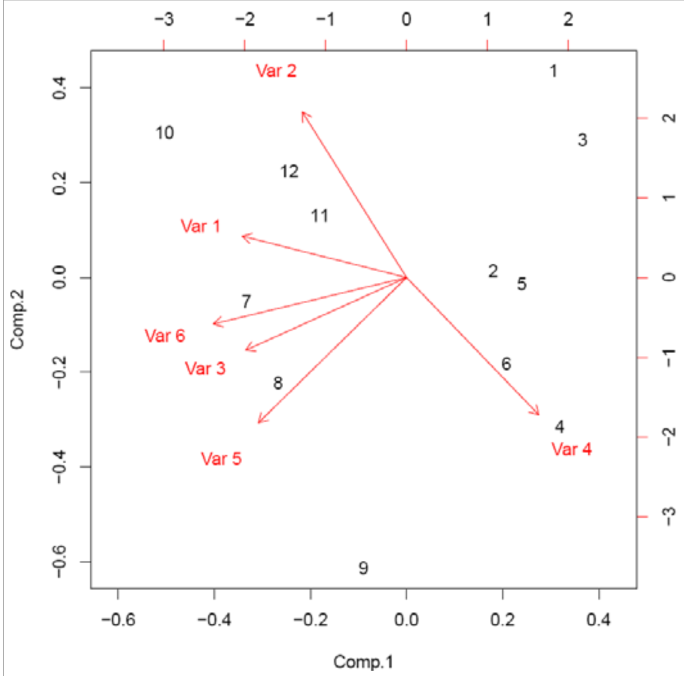

G Siberia Region HIV search biplot

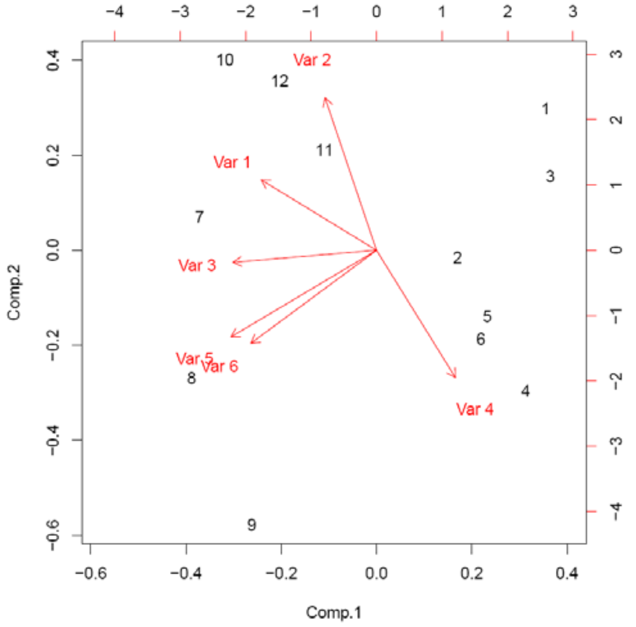

G Siberia Region HIV prevalence biplot

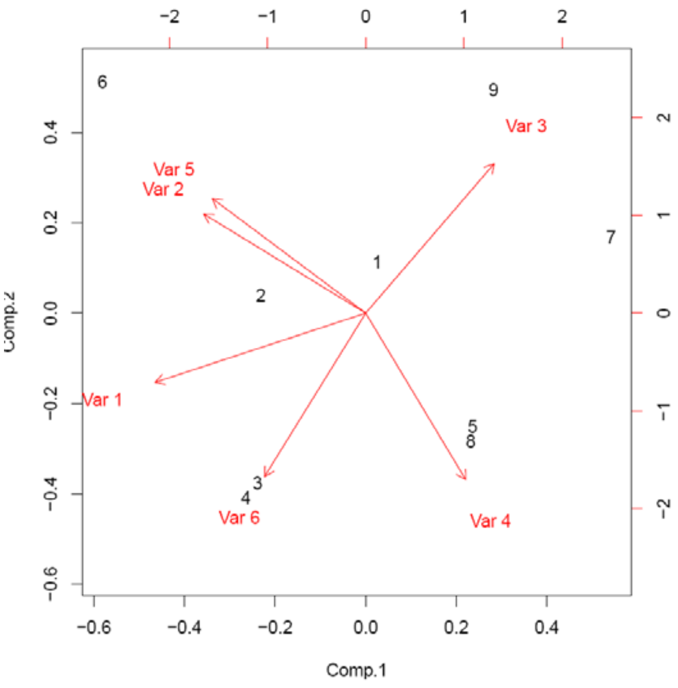

H Far East region HIV search biplot

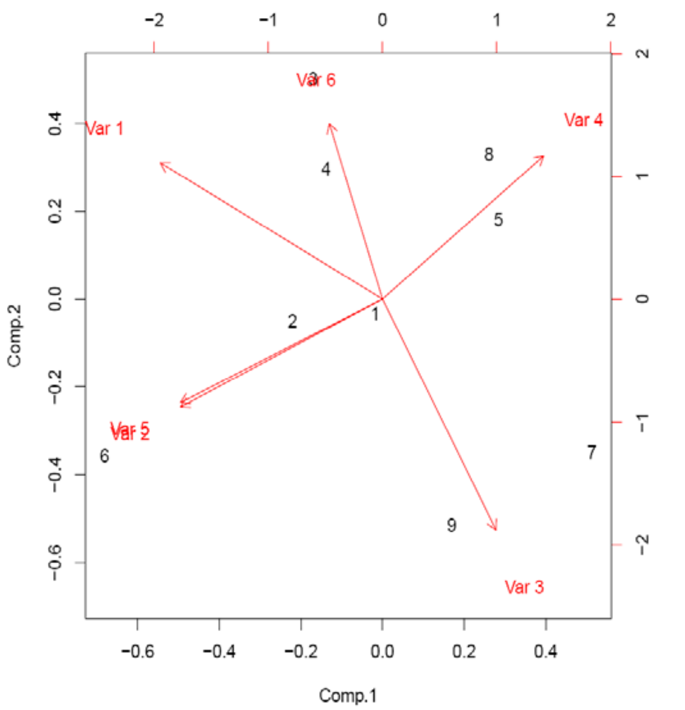

H Far East region HIV prevalence biplot
