# Supplementary material for: Internet Search Patterns of Human Immunodeficiency Virus and the Digital Divide in the Russian Federation: Infoveillance Study
Source: J Med Internet Res. 2013 Nov 12;15(11):e256. doi: 10.2196/jmir.2936 (PMC3841350; doi:10.2196/jmir.2936)
Supplement: Supplementary file 5 [file jmir_v15i11e256_app5.pdf]

Summary of regional PCA biplots results tables

Region	Relationship	Geographic clusters	Outliers
Central (A)	PC2:<br>V1, V3 V2,V6<br>Age, education, income and HIV prevalence/search	Cluster 1<br>A1. Belgorod<br>A2. Bryansk<br>A4. Voronezh<br>A8. Kursk<br>A9. Lipetsk<br>A11. Orel<br>A12. Ryazan<br>A14. Tambov<br><br>Cluster 2<br>A3. Vladimir<br>A5. Ivanovo<br>A6. Kaluga<br>A7. Kostroma<br>A10. Moscow Region<br>A15. Tver<br>A16. Tula<br>A17. Yaroslavl	A18. Moscow City
North West (B)	PC1:<br>V1, V2, V3, V6<br>Age, education, income HIV search/prevalence<br><br>PC2:<br>V4, V5<br>broadband fee, urbanisation	Cluster 1<br>B6. Kaliningrad<br>B7. Leningrad<br><br>Cluster 2<br>B1. Karelia<br>B2. Komi<br>B3. Archangelsk<br>B5. Vologda<br>B8. Murmansk<br>B9. Novgorod<br>B10. Pskov	B11.St Petersburg<br>B4. Nenets Autonomous Region
South (C)	PC1:<br>V4, V5<br>broadband fee, urbanisation,<br><br>PC2:<br>V1, V2, V3, V6<br>Age, Education, income, HIV prevalence/search	Cluster 1<br>C1. Adygia<br>C2. Kalmykia<br><br>Cluster 2<br>C4. Astrkhan<br>C6. Rostov	C3. Krasnodar<br>C5. Volgograd
North Caucuses (D)	PC2:<br>V1, V2, V3, V4, V5, V6	Cluster 1<br>D1.Dagestan<br>D3. Karbadino Balkaria<br>D4. Karachayevo cherkassia<br><br>Cluster 2<br>D2. Ingushetia<br>D6. Chechnya<br><br>Cluster 3<br>D5. North Ossetiya<br>D7. Stavropol	D7. Stavropol

Volga<br>(E)	PC1:<br>v1<br>Age<br><br>PC2: v2, v3, v4, v5, v6<br>Education, income, broadband fee, urbanisation, HIV prevalence/search	Cluster 1<br>E1. Bashkortostan<br>E2. Mariy El<br>E5. Udmutriya<br>E8. Kirov<br>E11. Penza<br>E14. Ulyanovsk<br><br>Cluster 2 “urban”<br>E4. Tatarstan<br>E9. Nizhegorod<br>E12. Samara<br>E13. Saratov<br><br>Cluster3 “rural”<br>E3. Mordovia<br>E6. Chuvashia<br><br>Cluster 4<br>E10. Orenburg<br>E7. Perm	E7. Perm<br>E12. Samara
Urals<br>(F)	PC1:<br>V5, V6<br>Urbanisation, HIV prevalence and search<br><br>PC2:<br>V1, V2, v3, v4<br>Age, education, income, broadband fee	Cluster 1<br>F3. Tyumen<br>F6. Chelyabinsk	F1. Kurgan<br>F5. Yamalo Nenets
Siberia<br>(G)	PC1: V2<br>Education<br><br>PC2: V1, v3, V4, v5, v6<br>Age, income, broadband fee, urbanisation, HIV search and prevalence	Cluster 1<br>G2. Buryatiya<br>G4. Khakasiya<br>G5. Altay<br>G6. Zabaykalya<br><br>Cluster 2<br>G10. Novosibirsk<br>G11. Omsk<br>G12. Tomsk<br><br>CLUSTER 3<br>G7. Krasnoyarsk<br><br>CLUSTER 4<br>G8. Irkutsk	G9. Kemerovo
Far East<br>(H)	PC1:v 3, v6<br>income and HIV prevalence/search<br><br>PC2: v1, v2, v4,v5<br>age, education, broadband fee, urbanisation	CLUSTER 1:<br>H5. Amursk<br>H8. Jewish Auton. Region.<br><br>CLUSTER 2:<br>H3. Primorsk<br>H4. Khabrovsk<br><br>CLUSTER 3:<br>H1. Sakha (Yakutia)<br>H2. Kamchatka<br><br>CLUSTER 4:<br>H7. Sakhalin<br>H9. Chukotka	H6. Magadan
